# RNA-Seq based transcriptome of whole blood from immunocompetent pigs (*Sus scrofa*) experimentally infected with *Mycoplasma suis* strain Illinois

**DOI:** 10.1186/s13567-018-0546-6

**Published:** 2018-06-18

**Authors:** Naíla C. do Nascimento, Ana M. S. Guimaraes, Andrea P. dos Santos, Yuefeng Chu, Lucas M. Marques, Joanne B. Messick

**Affiliations:** 1 Department of Comparative Pathobiology, College of Veterinary Medicine, Purdue University, West Lafayette, IN USA; 2 Department of Microbiology, Institute of Biomedical Science, University of São Paulo, São Paulo, Brazil; 3 Multidisciplinary Institute of Health, Federal University of Bahia, Vitória da Conquista, Bahia Brazil; 4 Present Address: Department of Microbiology, Institute of Biomedical Science, University of São Paulo, São Paulo, Brazil; 5 Present Address: State Key Laboratory of Veterinary Etiological Biology, Lanzhou Veterinary Research Institute of CAAS, Lanzhou, China

## Abstract

Pigs are popular animal models in biomedical research. RNA-Seq is becoming the predominant tool to investigate transcriptional changes of the pig’s response to infection. The high sensitivity of this tool requires a strict control of the study design beginning with the selection of healthy animals to provide accurate interpretation of research data. Pigs chronically infected with *Mycoplasma suis* often show no obvious clinical signs, however the infection may affect the validity of animal research. The goal of this study was to investigate whether or not this silent infection is also silent at the host transcriptional level. Therefore, immunocompetent pigs were experimentally infected with *M. suis* and transcriptional profiles of whole blood, generated by RNA-Seq, were analyzed and compared to non-infected animals. RNA-Seq showed 55 differentially expressed (DE) genes in the *M. suis* infected pigs. Down-regulation of genes related to innate immunity (*tlr8*, chemokines, chemokines receptors) and genes containing IFN gamma-activated sequence (*gbp1*, *gbp2*, *il15*, *cxcl10*, *casp1*, *cd274*) suggests a general suppression of the immune response in the infected animals. Sixteen (29.09%) of the DE genes were involved in two protein interaction networks: one involving chemokines, chemokine receptors and interleukin-15 and another involving the complement cascade. Genes related to vascular permeability, blood coagulation, and endothelium integrity were also DE in infected pigs. These findings suggest that *M. suis* subclinical infection causes significant alterations in blood mRNA levels, which could impact data interpretation of research using pigs. Screening of pigs for *M. suis* infection before initiating animal studies is strongly recommended.

**Electronic supplementary material:**

The online version of this article (10.1186/s13567-018-0546-6) contains supplementary material, which is available to authorized users.

## Introduction

The pig (*Sus scrofa*) is an important meat-production animal and the most commonly used large animal in research, particularly as a mammalian model for the study of human diseases [1]. Their size, anatomy, and physiology as well as the progression of many diseases, such as diabetes, atherosclerosis, and cardiovascular disease, are similar to humans [1]. Further, the improvement of techniques for gene modification has resulted in an increase in the number of genetically engineered swine models [2, 3]. Because of these factors, the National Institutes of Health (NIH) has established the National Swine Research and Resource Center to help meet the needs of the biomedical community [4].

The sequencing of the complete pig genome [5] confirmed that its gene content, sequence and chromosomal structure are highly conserved relative to the human genome [5, 6], making the pig a particularly useful model for genome-based studies. Accordingly, RNA sequencing (RNA-Seq) using next-generation sequencing (NGS) technology is becoming the predominant tool for large-scale gene expression analyses. Numerous studies using RNA-Seq methodology have been performed to investigate global gene expression in various tissues or cells from pigs of different breeds or phenotypes [7–14]. Since transcriptome analyses are extremely sensitive and specific, they may result in misleading and inaccurate functional genomic data if environmental and host factors are not tightly controlled in the study design. The selection of healthy pigs is the first step toward the goal of generating valid research data that will provide results that are more predictive of human diseases.

One of the most important host factors that may affect the validity of animal research is the presence of pre-existing subclinical infectious diseases in test and/or control animals. *Mycoplasma suis* is notorious for causing a chronic infection without obvious clinical signs. This organism typically attaches to porcine erythrocytes, and is the only hemotrophic mycoplasma shown to invade its host cell [15]. When chronically infected animals become stressed or undergo splenectomy, overt infection develops and they may succumb to an acute, life-threatening disease characterized by hemolytic anemia, hypoglycemia, and bleeding accompanied by intravascular coagulation and endothelial damage [16, 17]. Some reports also suggest that pigs chronically infected with *M. suis* may have impaired immune defenses, being more susceptible to respiratory and enteric diseases [18, 19]. Research pigs, however, are not routinely tested for the presence of *M. suis* in their bloodstream. We have shown that levels of bacteria may fluctuate from 10^3^ to 10^9^/mL of blood for months in naturally infected pigs [20]. Considering the high within-herd prevalence (up to 86%) and wide geographic distribution [21–23], *M. suis* constitutes a major threat to the use of pigs in research.

Hemotrophic mycoplasmas, such as *M. suis*, have been described in several mammalian species and the recrudescence of acute disease in chronically infected animals, especially when stressed, has been reported to have a deleterious effect on the course of experimental research in splenectomized pigs and dogs, non-human primates (*Saimiri sciureus*), rodents, and sheep [24–28]. These findings strengthen our hypothesis that chronic infections, while clinically silent, are unlikely to be silent at the host transcriptional level and should be further explored. Therefore, experimental *M. suis* infection of immunocompetent (non-splenectomized) pigs, a model for mimicking natural subclinical infection, was performed to study its effects on the blood transcriptional profile of the porcine host.

## Materials and methods

### Animals

The Purdue Animal Care and Use Committee, Protocol Number 1111000223, approved this study. Six domestic piglets (*Sus scrofa domesticus*), all females from the same litter, were purchased from the Animal Sciences Research and Education Center-ASREC, Purdue University, West Lafayette, IN, USA. The animals were received when they were 20 days old and kept until turning 99 days old at Purdue animal housing facilities. Piglets were fed antibiotic-free feed, as well as water ad libitum for the entire study.

### Bacterial strain

The *M. suis* strain Illinois, obtained in 1985 from a naturally infected pig having acute infection and high loads of bacteremia, was used in an experimental infection study [18]. After 14 years of storage (−80 °C), *M. suis* strain Illinois, as demonstrated by experimental infection of pigs, retained its pathogenicity [29]. Aliquots collected from a specific pathogen free (SPF) pig inoculated with *M. suis* and at the peak of bacteremia [29], representing the second passage of this strain, were frozen at −80 °C and used in the experimental infection described herein.

### Study design and experimental infection

Piglets were considered *M. suis*—free by testing blood samples using qPCR [20] 13 days after birth at ASREC and twice (at 33 and 53 days old) prior to the infection day while at Purdue animal housing facilities. The mother also tested negative for *M. suis* by qPCR while pregnant, 50 days prior to farrowing. They were randomly assigned to either a control group (*n* = 3; pigs #1, #2, and #3) or an experimental *M. suis* infection group (*n* = 3; pigs #5, #6, and #7). The control group was housed in a separate room from the infection group to avoid *M. suis* transmission. Thirty-three days after arrival (day 0), experimental infection was performed. Briefly, animals were sedated with TX (telazol reconstituted with 500 mg of xylazine, 1.0 mL/100 kg of body weight); three piglets in the infection group were intravenously inoculated through the ear vein with 1.0 mL of cryopreserved blood containing *M. suis* strain Illinois at a concentration of 10^8^ to 10^9^ organisms/mL of blood. After inoculation, 1.0 mL of heparin sodium was flushed through the butterfly catheter. Pigs in the control group (sham-infected) were also intravenously inoculated, using 1.0 mL of blood collected from another control pig, collected at the same time. The animals were monitored daily (minimum twice a day) for direct observation of behavior (BAR *status*) and body temperature. Blood was collected through the anterior venous cava vein and added to EDTA tubes every 3–4 days for monitoring infection by qPCR [20], hematocrit, and detection of specific antibodies for *M. suis* recombinant GrpE (rGrpE) antigen using microbead immunoassay (MIA) [30]. Pigs were considered seropositive when their median fluorescence intensity (MFI) crossed the cut-off defined as the mean plus 3 standard deviation of the pre-inoculation MFIs (day 0) for all animals.

### RNA preparation and RNA-sequencing (RNA-Seq) procedure

Six aliquots of 400 L EDTA-whole blood from each animal were immediately transferred after collection to 2 mL tubes containing TRIzol^®^ reagent (Life Technologies Corp., Carlsbad, CA, USA) and stored at −80 °C until RNA extraction. Total RNA was extracted using TRIzol^®^ (Life Technologies Corp.) followed by RNeasy Mini Kit, including on-column DNase treatment step (QIAGEN^®^ Inc., Valencia, CA, USA) [13]. Concentration and quality of the RNA samples were assessed using Agilent 2100 Bioanalyzer (Agilent Technologies, Inc. Headquarters, CA, USA), and samples with highest RIN (RNA integrity number) and concentration were chosen for mRNA purification. Enriched mRNA (purified with Globin-Zero™ Gold Kit, Epicentre^®^, by Illumina^®^ company, Madison, WI, USA) was sent to the Genomics Core Facility at Purdue University for library construction and RNA-sequencing. One RNA sample from each pig was selected for transcriptome analysis based on the first, highest load of *M. suis* detected (peak of bacteremia; shown in results) in the peripheral blood of the infected pigs according to qPCR results. The days selected for the infected group were: pig #5, 30 days post-inoculation (dpi); pig #6, 23 dpi, and pig #7, 23 dpi; the highest peaks of bacteremia for each animal within 7–8 days from each other. For control pigs (#1, #2, and #3), 27 dpi was chosen for transcriptome analysis, as an intermediate day among days 23 and 30. Samples were sequenced from paired-end libraries (TruSeq DNA sample preparation kit, Illumina Inc., San Diego, CA, USA) barcoded with different tags using one lane of Illumina HiSeq 2000.

### Differential gene expression analysis of pigs infected with *M. suis* and control pigs

Illumina reads were pre-processed for mapping by assessing quality using FastQC (v 0.10.1) [31], and trimming was done using FASTX toolkit (v 0.0.13). The quality trimmed reads were mapped against the bowtie2-indexed *Sus scrofa* genome (version 75 from ENSEMBL) using Tophat (v 2.0.9) [32] with default parameters. HTSeq (v 0.5.3p7) was used to generate raw read counts from each sample for each gene feature using Tophat output and the known *Sus scrofa* annotations. Genes with zero counts were removed from the count matrix and genes where the count was zero in some samples and not in others were changed to one in order to prevent infinite values during the statistical analysis.

Three different methods were used to perform pairwise analyses of differential gene expression between control and *M. suis*-infected groups. In the first two methods, edgeR (v 3.0.3) and DESeq 2 (v 1.0.19), “R” package (Version 2.15.2) [33] was used to statistically analyze the results based on the count matrix mentioned above. And finally, in the third method (Cufflinks), bam files from mapping to the genome were analyzed using the Cufflinks (v 2.0.2) suite of programs (Cufflinks, Cuffmerge and Cuffdiff) [34]. The lists of significant differentially expressed (DE) genes were generated by using the adjusted *p*-value or FDR values. DE genes identified by at least two methods (edgeR, DESeq 2 and Cufflinks) (cut-off of *p*-value ≤ 0.1) were defined as truly DE genes.

### Quantitative RT-PCR validation of selected genes

Selected up and down DE genes were validated using two step quantitative RT-PCR (qRT-PCR). Briefly, independent cDNA synthesis was performed for all samples (three infected and three control pigs) starting from 100 ng of total RNA, same total RNA used for RNA-Seq procedure, using SuperScript^®^ VILO™ cDNA Synthesis Kit (Invitrogen, Carlsbad, CA, USA). qPCR was carried out in a 7300 Real-Time PCR System (Applied Biosystems^®^, Foster City, CA, USA) using Power SYBR^®^ Green PCR Master Mix (Applied Biosystems^®^) as chemistry according to manufacturer’s instructions. Primers for each target gene selected for validation by qRT-PCR, in addition to *GAPDH*, used as reference gene, were designed using Primer3web version 4.0.0 [35] (Table 1). In addition to the biological replicates (cDNA from three infected and three control pigs) three technical replicates for each target gene (DE gene selected from RNA-Seq results) were included in the qPCR assay. *GAPDH* replicates were added and analyzed in every assay for correct comparison of target genes between infected and control pigs. Relative quantification was evaluated using the Comparative Ct method with *GAPDH* gene as reference gene [36]. Fold change was calculated as the difference between infected and control group. Graphics of relative expression display the mean ± standard deviation of biological replicates.

**Table 1:** **Primers used for validation of differentially expressed (DE) genes in** ***Mycoplasma suis*** **-infected pigs by quantitative reverse-transcriptase polymerase chain reaction (qRT-PCR)**

	Gene symbol	Gene description	Primer forward (5′–3′)	Primer reverse (5′–3′)	Amplicon length (bp)
Reference gene	*GAPDH*		TTGGCTACAGCAACAGGGTG	CAGGAGATGCTCGGTGTGTT	166
DE genes	*ISG15*	ISG15 ubiquitin-like modifier	ATGTGCTTCAGGATGGGGTG	AGGATGCTCAGTGGGTCTCT	100
	*IL22RA2*	Interleukin 22 receptor, alpha 2	ACCAGCAACAGCAGCATCTA	TGAGTGCATCCCAGCCAAG	177
	*CD274*	CD274 molecule	TTACCCAGAGGCCGAAGTCA	TCCTCTCTCTGGGAACTGGT	87
	*BCL2L14*	BCL2-like 14 (apoptosis facilitator)	TGCCTACAGGGTTCCGTTTC	TTCGTGGTCTAAGCGCTGTT	113
	*CCR5*	Chemokine (C-C motif) receptor 5	CAGTGGGTCTAACAGGCTGG	GCGTCTGACGATGTGCTTTC	151
	*CD180*	CD180 molecule	TGCTCATCGTCCTGCTCATTT	CCAGCTCCAGGAACCAATCT	178
	*AIF1*	Allograft inflammatory factor 1	GCGAGAGAACAGGAAAAGCC	AGCCCCTTCAATTCCACCAC	100
	*IL15*	Interleukin 15	GCTCATCCCAATTGCAAAGT	TTCCTCCAGCTCCTCACATT	189 [37]
	*PTPRO*	Protein tyrosine phosphatase, receptor type, O	AAGCACCAGGACGACTTAGC	AACCCCAAAACTCAGCCCAA	174
	*TLR8*	Toll-like receptor 8	CGGAAGGCTTGTTTTGGCAA	CGACCAAACATCACCGAGGA	112

### Statistical analysis

The relative expression of the target genes tested by qRT-PCR between infected and control groups was compared by *t*-test (*p*-value ≤ 0.1) using JMP^®^ Software (JMP^®^, SAS Institute Inc., Cary, NC, 1989–2007).

### Network associations and gene ontology annotation of DE genes

DE genes identified in *M. suis* infected pigs were analyzed using STRING database with default settings for prediction of network associations between proteins [38]. In addition, Blast2Go software was used to assign functional categories to each protein encoded by DE genes based on gene ontology (GO) terms [39].

## Results

### Hematocrit, rectal temperature, *M. suis* loads, and humoral response to rGrpE

**Figure 1:**
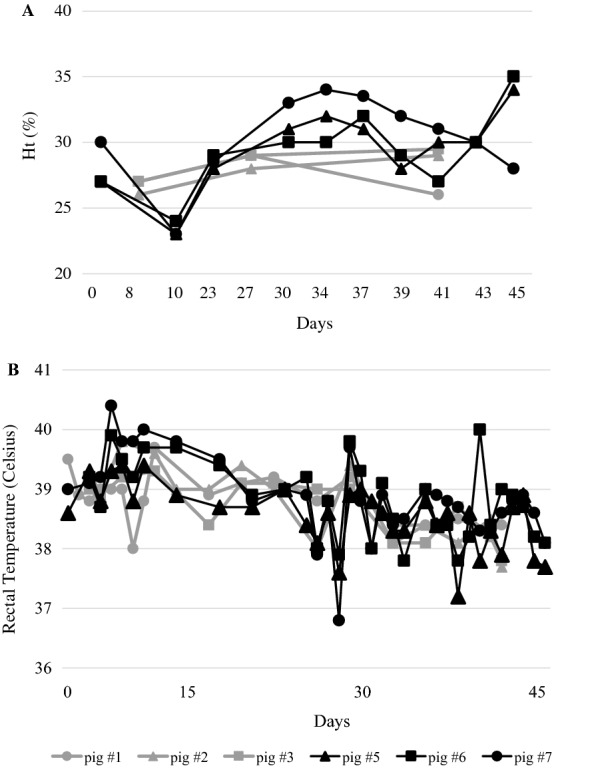
**Hematocrit (Ht) and rectal temperature of** ***Mycoplasma suis*-infected and sham-inoculated pigs. A** Hematocrits were determined for each pig along the course of the experiment. **B** Rectal temperature was measured for each pig along the course of the experiment. *M. suis*-infected group: pig #5, pig #6, pig #7. Sham-infected group: pig #1, pig #2 and pig #3. Day 0 corresponds to inoculation day.

Results of hematocrit and rectal temperature of *M. suis*-infected and sham-inoculated groups are shown in Figure 1. The hematocrit of *M. suis* infected pigs ranged from 23 to 35%, while in sham-inoculated animals this number varied from 26 to 29% (Figure 1A). Interestingly, the lowest hematocrit values (pig #5: 23%; pig #6: 24%; pig #7: 23%) were observed 10 dpi in *M. suis* infected-pigs. The rectal temperature of *M. suis*-infected and sham-infected pigs ranged from 36.8 to 40.4 °C and from 37.7 to 39.8 °F, respectively (pig reference interval 38.7–39.8 °C) (Figure 1B). High rectal temperatures (> 39.8 °C, maximum of 40.4 °C) were observed intermittently during the first 7 dpi only in pigs #6 and #7 from the *M. suis*-infected group (Figure 1B). Despite these minor increases in rectal temperatures of *M. suis*-infected pigs, none of the six animals displayed obvious clinical signs of infection.

**Figure 2:**
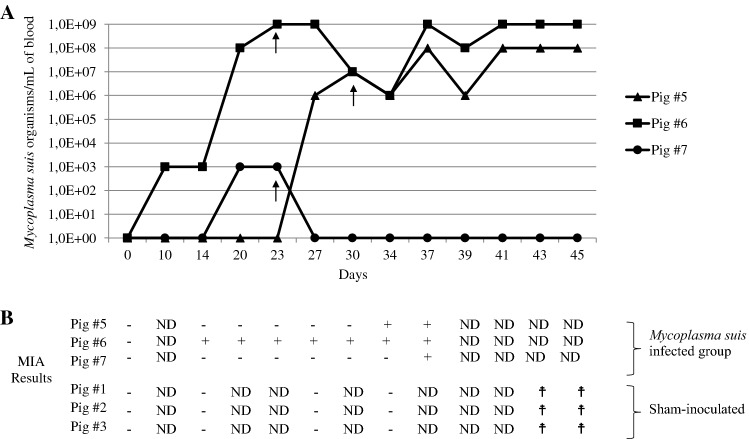
***Mycoplasma suis*** **loads and MIA (microbead immunoassay) results of** ***Mycoplasma suis*-infected and sham-inoculated pigs. A** The copy number of *M. suis* strain Illinois organisms was detected by qPCR [20] on blood samples of *M. suis*-infected pigs (pig#5, pig#6 and pig#7) along the course of the experiment. Sham-inoculated pigs (pig #1, pig #2 and pig #3) were consistently negative on qPCR for the extent of the experiment. **B** Serum antibodies were measured using recombinant GrpE (rGrpE) protein in a microbead immunoassay (MIA) [30]. + = positive, − = negative, based on established MFI (median fluorescence intensity) cut-off. ND: not determined. ^☨^pigs have already been euthanized. Serum MFI values above the established cut-off values were considered positive, while serum MFI values below the established cut-off values were considered negative. MFI cut-off for all pigs was 74.07. It is important to note that a high cut-off may have been encountered due to the low number of animals to determine a cut-off, which increases the standard deviation. *M. suis*-infected group: pig #1, pig #2 and pig #3. Sham-inoculated group: pig #5, pig #6, pig #7. Day 0 corresponds to inoculation day. At day 0, blood was collected prior to *M. suis* or sham-inoculation. Black arrows indicate the days chosen for RNA-Seq analysis for each pig.

Results of *M. suis* copy number/mL of blood and humoral response (MIA) of *M. suis*-infected and sham-inoculated pigs are shown in Figure 2. All three pigs inoculated with *M. suis* strain Illinois became infected (as shown by qPCR results) and seroconverted (as shown by MIA results). Infected pigs showed the first qPCR positive result for *M. suis* on different days following experimental infection, varying from day 10 (pig #6) to day 27 (pig #5). Bacterial loads varied from approximately 10^3^ to 10^9^ organisms/mL of blood. Interestingly, pig #7 was qPCR positive for *M. suis* on only two separate occasions, having a relatively low bacterial load (10^3^ organisms/mL of blood). According to the established MFI cut-off (74.07), all three pigs (infected group) seroconverted by days 14 (pig #6), 34 (pig #5) or 37 (pig #7) post inoculation and remained seropositive (pigs #5 and #6) until the last day evaluated (37 dpi) (Figure 2; MFI values varied from 20.5 to 939; individual MFI values are given in Additional file 1). It is important to note that, although pigs #5 and #7 seroconverted at a later day post-inoculation, both animals showed a constant increase on their individual MFI values at earlier days post-inoculation (Additional file 1), which likely represents earlier seroconversion. Sham-infected animals remained *M. suis* qPCR and MIA negative throughout the experiment (MFI values varied from 25.5 to 58.2; Additional file 1).

### RNA-Seq results and validation by qRT-PCR

**Figure 3:**
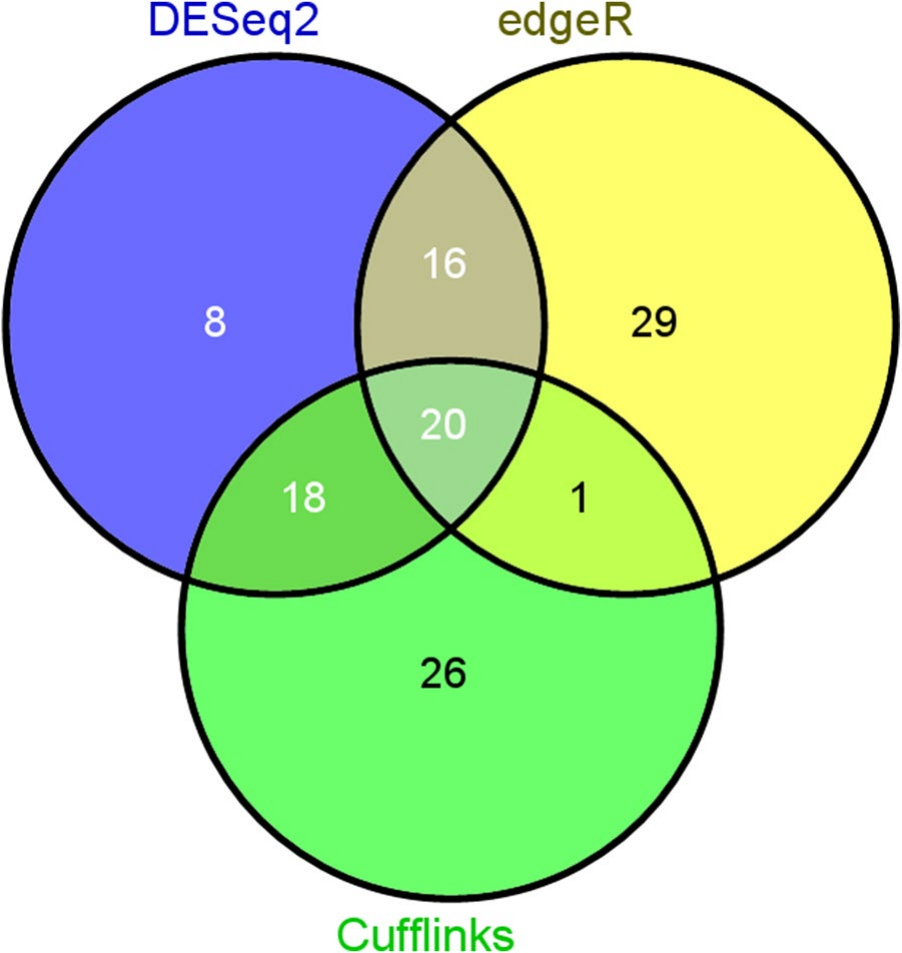
**Venn diagram of RNA-Seq differentially expressed (DE) genes (up and down-regulated) identified in the** ***Mycoplasma suis*-infected pigs group by three methods of pairwise differential expression analysis.** A total of 118 DE genes (20 up and 98 down-regulated) were identified by at least one of the three methods (Cufflinks, DESeq2, and edgeR) used for the RNA-Seq data analysis (cut-off of *p*-value ≤ 0.1).

Total RNA concentrations and quality of the samples were as follows: pig #1, 156.5 ng/µL, RIN 8.8; pig #2, 77.7 ng/µL, RIN 8.5; pig #3, 107.9 ng/µL, RIN 8.9; pig #5, 113.1 ng/µL, RIN 8; pig #6 30.9 ng/µL, RIN 8; pig #7 115.7 ng/µL, RIN 8.5. A Venn diagram showing the number of DE genes identified (*p*-value ≤ 0.1) in the *M. suis*-infected group when compared to the sham-inoculated group by each of the three analysis methods (DESeq 2, edgeR and Cufflinks) is shown in Figure 3. A list of all DE genes and their respective fold change and p-values are available in Additional file 2. Twenty DE genes were detected by all three methods, while 55 DE genes, 3 up and 52 down-regulated, were identified by at least two of the three methods (*p*-value ≤ 0.1) (Figure 3). Forty-seven out of the 55 DE genes (85.45%) were predicted to encode proteins with assigned functions, while 8 (14.55%) encode proteins with uncharacterized functions (Additional file 2). The raw RNA-Seq data was submitted to the Sequence Read Archive (SRA) database at the National Center for Biotechnology Information (NCBI) under Bioproject Accession Number PRJNA321932.

Approximately 20% of the 55 DE genes (*n* = 10) were chosen for qRT-PCR validation based on their role in immune response and/or fold change of 2× or more (Table 2). The relative gene expression results (mean and standard deviation) of DE genes validated by qRT-PCR are shown on Additional file 3. All genes were confirmed to be up or down regulated based on qRT-PCR results (*p*-value ≤ 0.1) (Table 2).

**Table 2:** **Differentially expressed (DE) genes identified in** ***Mycoplasma suis*** **-infected pigs tested by quantitative reverse-transcriptase polymerase chain reaction (qRT-PCR)**

	Gene ID	Gene symbol	Description	qRT-PCR FC	RNA-Seq FC^a^	qRT-PCR *p*-value	RNA-Seq *p*-value**
DE up-regulated genes	100145895	*ISG15*	ISG15 ubiquitin-like modifier	2.79	4.72	0.07	0.04
DE down-regulated genes	100513324	*IL22RA2*	Interleukin 22 receptor, alpha 2	0.01	0.02	0.03	0.01
	574058	*CD274*	CD274 molecule	0.11	0.17	0.01	0.01
	100514901	*BCL2L14*	BCL2-like 14 (apoptosis facilitator)	0.24	0.25	0.01	0.07
	414371	*CCR5*	chemokine (C–C motif) receptor 5	0.37	0.33	0.04	0.03
	397644	*CD180*	CD180 molecule	0.37	0.26	0.01	0.02
	397271	*AIF1*	Allograft inflammatory factor 1	0.19	0.20	0.00	0.01
	397683	*IL15*	Interleukin 15	0.22	0.24	0.03	0.02
	100524563	*PTPRO*	Protein tyrosine phosphatase, receptor type, O	0.40	0.28	0.01	0.01
	397384	*TLR8*	Toll-like receptor 8	0.40	0.32	0.04	0.05

FC: fold change of a DE gene in the infected group related to the control group, *GAPDH* was used as reference control gene for calculation of relative quantification.

** *p*-values based on Cufflinks results. Note that a FC of 0.01 of a down-regulated gene indicates that infected group has 1/100 the amount of target RNA as the control group.

^a^FC based on Cufflinks results.

### Network analysis of the relationships between proteins encoded by DE genes

**Figure 4:**
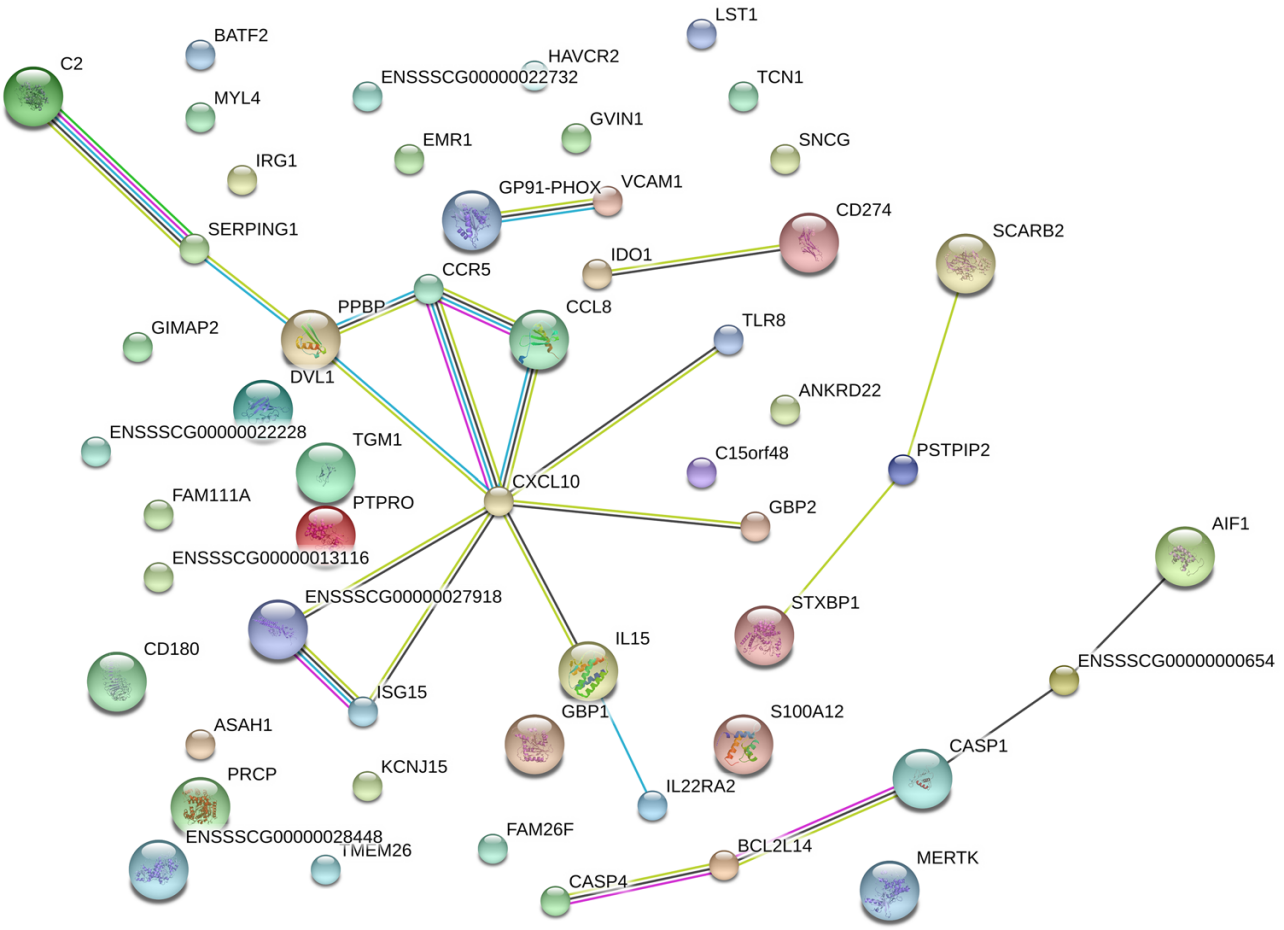
**STRING network analysis of the relationship between proteins encoded by differentially expressed (DE) genes identified in** ***Mycoplasma suis*-infected pigs.** The DE genes in whole blood of *M. suis*-infected pigs were analyzed using the STRING database [38]. The network nodes represent the proteins encoded by the DE genes. Seven different colored lines link a number of nodes and represent seven types of evidence used in predicting associations: green lines represent neighborhood evidence; red lines indicate the presence of fusion evidence; blue lines represent co-occurrence evidence; black lines represent co-expression evidence; purple lines represent experimental evidence; light blue lines represent database evidence; and yellow lines represent text-mining evidence.

STRING database predictions of functional association networks for all 55 proteins encoded by DE genes identified by 2 or more methods are shown in Figure 4. A main network involving chemokines, chemokine receptors and interleukin-15 was observed (encoded by genes *CXCL10*, *CCL8*, *PPBP*, *CCR5* and *IL15*). This network is part of pathways ID GO:0006954 and GO:0006955 (biological process, gene ontology), and 04060 and 04062 (KEGG pathways), corresponding to inflammatory response, immune response, cytokine–cytokine receptor interaction, and chemokine signaling pathways, respectively. Interactions among genes involved in programmed cell death (CASP4, BCL2L14 and CASP1) were also noted. Interestingly, genes containing IFN gamma-activated sequence (GAS) (e.g. *GBP1*, *GBP2*, *IL15*, *CXCL10*, *CASP1*, and *CD274*) are scattered throughout different networks, and a strong interaction between C2, the gene encoding for Complement 2 protein, and the C1 inhibitor (C1-INH, encoded by *SERPING1* gene), both involved in the complement cascade, was detected. Further, many proteins (28/55, 50.9%) that showed no direct relationships to other proteins were also identified in the analysis.

### Gene ontology analysis of differentially expressed (DE) genes of *M. suis* infected pigs

DE genes of *M. suis* infected pigs were analyzed using Blast2GO bioinformatics tool, which provides functional annotation of sequences by mapping the Blast hits of each sequence with gene ontology terms assigning molecular functions, biological processes and/or cellular components to each sequence. A total of 314 different functional categories, identified as biological processes, were assigned to the proteins encoded by DE genes (Additional file 4). Many proteins were assigned more than one “biological processes” roles. The top five identified functions under the “biological processes” category are listed on Table 3, which includes 18 of the 55 (32.7%) identified genes that were DE by 2 methods or more. These 5 functions of the “biological processes” category had at least 4 genes each. The following GO categories were also important (representing 3 or 4 genes each): chemokine-mediated signaling pathway; chemotaxis defense response to virus; G-protein coupled receptor signaling pathway; ion transport; negative regulation of T cell proliferation; oxidation–reduction process; positive regulation of T cell proliferation; protein phosphorylation; regulation of apoptotic process; response to lipopolysaccharide; transport; signal transduction (*n* = 3 each); ion transport (*n* = 4). These functional roles indicate that DE genes were mostly associated with immune and inflammatory responses.

**Table 3:** **Four main gene ontology (GO) functions, identified as biological processes, of differentially expressed (DE) genes of** ***Mycoplasma suis*** **infected pigs**

Biological process^a^	Number of DE genes	Gene symbol^b^
Inflammatory response	9	*CCR5*, *CYBB*, *IDO1*, *TLR8*, *CASP4*, *CASP1*, *CXCL10*, *AIF1*, *CD180*
Immune response	5	*CCR5*, *TLR8*, *CCL8*, *CXCL10*, *PDL1*
Apoptotic process	5	*CASP4*, *BCL2L14*, *CASP1*, *AIF1*, *MERTK*
Proteolysis	6	*C2*, *C1*-*INH*, *CASP4*, *CASP1*, *LAP3*, *PRCP*
Cellular response to lipopolysaccharide	4	*CCR5*, *HAVCR2*, *CXCL10*, *CD180*

^a^Biological processes were identified by Blast2GO bioinformatics tool [39].

^b^Descriptions of each gene are listed on Additional file 4.

## Discussion

In this study, immunocompetent pigs experimentally infected with *M. suis* did not show clinical signs of overt acute disease during the whole experiment, except for mild, transient temperature elevations and low hematocrit values (23–24%) at 10 dpi. Thus, a subclinical infection, resembling the chronic type of the disease seen in the field, developed, with bacteremia first detected by qPCR between days 10 and 27 post *M. suis* inoculation. It is unclear whether one pig (pig #7) eliminated the infection after day 23 or whether its bacterial loads fell below the detection limit of our assay. This variability in the course of *M. suis* infection may have important implications related to the host–pathogen interactions identified herein. Nevertheless, the transcriptome profile obtained in this study was performed on samples collected at the first highest level (peak) of bacteremia for each individual animal within the same week, which may mitigate possible biases. The study reveals that pigs subclinically infected with *M. suis* show altered patterns of gene expression when compared to non-infected pigs, confirming our main hypothesis.

Immune mediated hemolytic anemia (IMHA) has been described in *M. suis*-infected pigs. Reactive antibodies can be detected as early as 10 days following *M. suis* inoculation in splenectomized pigs [40]. The low hematocrit values (23–24%) detected 10 dpi in the infected pigs may be a result of a *M. suis*-induced IMHA. It is unlikely a direct consequence of the pathogen, which was still undetectable by qPCR. This finding warrants further studies in the pathophysiology of *M. suis*-induced IMHA in non-splenectomized pigs and its impact on pig health.

Pigs #5 and #6 showed consistently high *M. suis* blood loads (between 10^8^ and 10^9^ organisms/mL of blood) and showed no clinical signs or significant alterations in hematocrit values during these “peaks” of bacteremia. A similar phenomenon (up to 8.23 × 10^8^ organisms/mL of blood, no anemia and negative blood smears) has been observed in a naturally infected, non-splenectomized pig that was followed-up for 80 days by our research group [20]. In that same study, a splenectomized pig showed profound anemia when *M. suis* loads reached between 10^11^ and 10^12^ organisms/mL of blood, which were easily detected on blood smears. On the other hand, anemia and a significant inverse correlation between *M. suis* loads in the blood and hematocrit values have been described in non-splenectomized, feeder pigs from Germany that have showed up to approximately 10^9^ organisms/mL of blood [22]. The discrepancy in blood loads in anemic and non-anemic animals observed in different studies may be due to differences in *M. suis* PCR detection protocols and has been previously discussed [20]. Nevertheless, the extent of individual hematological parameter variations of pigs with high *M. suis* loads (> 10^8^ organisms/mL of blood), and the influence of stress, splenectomy, genetics, co-infections and age on *M. suis* infection have never been fully explored. Whether or not some of these factors have affected the clinical course of *M. suis* infection in the studied pigs or in pigs from different studies is unknown.

Due to recent advances in NGS platforms, RNA-Seq studies have become a cost-effective and efficient approach to study global gene expression based on RNA levels within a biological sample. Unfortunately, there is no consensus on which analysis method should be used to guarantee maximum sensitivity and specificity in the detection of DE genes. A recent study that compared the performance of the three most frequently used software tools for RNA-Seq analysis (Cufflinks, DESeq and edgeR) identified that edgeR performs slightly better than the other two methods to detect true positives (i.e. truly DE genes) [41]. Nevertheless, in order to decrease the possibility of false positives, it is still recommended that DE genes should be detected based on their identification in two or more analysis tools [41]. Considering that the objective of our study was to determine if *M. suis* infection truly causes alterations in pigs’ gene expression profiles, we considered only genes that were identified as DE by two or more methods, thus decreasing the chance of detecting false positive DE genes.

As whole blood (i.e. multiple cell types) was the RNA source of this study, comparisons regarding the detected DE genes with studies using the same system in pigs are limited. However, the significance of each detected DE gene for the pathogenesis of the disease can be evaluated using information available in the literature about the expected function of these genes in each white blood cell (e.g. lymphocytes, monocytes, neutrophils). Interestingly, most DE genes in this study were down-regulated. While several mycoplasma infections are associated with up-regulation of pro-inflammatory genes related with overt disease [42–44], our study focused on subclinical infection; thus, more subtle changes were expected. A general suppression of genes related to the innate immune response was observed in the *M. suis*-infected animals. Accordingly, the expression of *TLR8* (Toll like receptor 8) and *CD180* genes, the physiological inhibitor of TLR4, was down-regulated in infected pigs. The activation of TLRs by pathogen associated molecular patterns (PAMPs) is critical to the induction of an innate immune response. TLR8 recognizes viral or bacterial single-stranded RNA and is primarily expressed in monocytes/macrophages on intracellular vesicular membranes [45–49]. On the other hand, CD180 expressed in monocytes/macrophages, inhibits TLR4 signaling and thus, low levels can increase TLR4 mediated inflammatory response upon stimulation [50]. *TLR4* mRNA expression, however, was not elevated in *M. suis* infected pigs despite the down-regulation of CD180. Another way by which *M. suis* may be modulating the innate response is through the down-regulation of cytochrome b-245 beta polypeptide (*CYBB*) and indoleamine 2,3-dioxygenase (*IDO1*) genes. CYBB is part of the NADPH oxidase 2 enzyme (NOX2) responsible for the production of superoxide in phagocytes. Low levels of NOX2 may dampen the microbicidal activity of these cells [51]. IDO1 is a tryptophan-degrading enzyme mainly present in antigen presenting cells (APC). It can have opposing effects against intracellular pathogens by either suppressing or increasing their replication [52]. Thus, the down-regulation of *TLR8*, *CD180*, *CYBB* and *IDO1* suggests mechanisms by which *M. suis* may modulate innate immune responses. Considering that many biomedical research projects using pig models focus on the evaluation of innate immune responses, alterations in such genes are likely to interfere with subsequent research results.

The biology of inflammasomes and programmed cell death has been increasingly evaluated in biomedical research projects. The observed down-regulation of the genes that encode caspase-1 (*CASP1*), caspase-4 (*CASP4*), apoptosis facilitator Bcl-2-like protein 14 (*BCL2L14*), and programmed death-ligand 1 (*PD*-*L1* or *CD274*) suggest the possibility that *M. suis* is able to interfere with the host’s programmed cell death. Pyroptosis, a type of programmed cell death, is a caspase-1-mediated process that drives the host response to an inflammatory state through the recruitment of the inflammasome’s multiprotein oligomers [53]. Caspase-4 has also been linked to inflammasome signaling involving caspase-1-dependent and caspase-1-independent outcomes [54–56]. Intracellular pathogens and LPS have been described to directly interfere with these caspases [56–58]. BCL2L14 protein, on the other hand, are linked to the p53-dependent intrinsic apoptosis pathway [59]. Whether or not *M. suis* may be acting to inhibit the programmed cell death of peripheral blood leukocytes needs to be further explored.

Cytokine production has been studied in porcine models of infectious and metabolic diseases for many years. Numerous commercial assays exist to measure cytokines and chemokines in pig samples. Herein, the down-regulation of several IFN-γ-regulated genes (a type II-IFN) could indicate a mechanism of impaired IFN-γ control of *M. suis* infection, which could directly interfere with cytokine analyses of pigs in research studies if animals are infected. It is important to note that IFN genes were not differentially expressed in our study, however only one time point was evaluated and the expression of these genes could have varied over the course of infection. The only interleukin gene with altered mRNA levels in *M. suis* infected pigs was *IL15*, which was down-regulated. Deficiency of IL15 may enhance erythropoiesis [60], but at the same time hamper the maintenance of memory CD8+ T cells and NK proliferation [61]. The failure in maintaining proper levels of memory CD8+ T cells may contribute to impaired host protection to re-infection. While cats can be resistant to re-infection with *M. haemofelis* [62], this feature is still unclear for *M. suis*.

The bradykinin, complement, coagulation and fibrinolytic cascades play important roles in inflammation [63, 64]. Of particular interest, C1 inhibitor (C1-INH, encoded by *SERPING1* gene), which was down-regulated in *M. suis* infected pigs, is a major biological inhibitor regulating several proteins of these pathways. The biological activities of C1-INH can be broadly divided into regulation of vascular permeability, coagulation and anti-inflammatory functions [65]. The clinical manifestations of the C1-INH deficiency are dramatically demonstrated in hereditary angioedema (HAE) in humans, where the principle mediator of subcutaneous and submucosal angioedema is excessive bradykinin production [63]. Vascular edema has been suggested to occur in pigs infected with *M. suis* [16] and Arthus-like vascular lesions have been reported in cattle infected with *M. wenyoni*, another hemotrophic mycoplasma [66]. In addition, intravascular thrombosis, superficial hemorrhage (petechia and ecchymosis), prolonged partial thromboplastin and prothrombin times, and decreased platelet count have been observed in splenectomized *M. suis*-infected pigs [16, 17, 67]. Although other molecules are likely involved in the disturbance of hemostasis in acute infection, the absence of C1-INH could establish a pro-coagulation and pro-thrombotic state in subclinical infections. This could more easily trigger the coagulation cascade leading to clot formation and contributing to angioedema formation by increasing vascular permeability [68]. Although *M. suis* chronically infected animals show a less severe disease when compared to acutely infected pigs [17], transcriptional changes observed herein suggest mechanisms that help explain clinical signs and laboratory findings observed during acute attacks, as well as the host’s impaired ability to stop the development of intravascular coagulation. While it is possible that these lesions are related to deficiencies of C1-INH, further studies are needed to clearly define the mechanism involved.

Surprisingly, several DE genes related to endothelial cell physiology and angiogenesis were identified in our analyses. The expression of the gene encoding plasma prolylcarboxypeptidase (*PRCP* or *angiotensinase C*), a membrane protein of endothelial and other cells that cleaves a variety of bioactive peptides, including bradykinin, angiotensin II/III, and prekallikrein, was down-regulated at the time point evaluated during subclinical *M. suis* infection. PRCP activity also has been identified in blood leukocytes in humans, principally monocytes/macrophages [69]. It has been reported that this serine protease acts like an endothelial growth factor. A series of experiments has shown that PRCP deficiency in mice is associated with decreased endothelial cell growth, reduced angiogenesis and impaired wound healing following vascular injury [70]. Given the recently recognized cellular tropism of *M. suis* for vascular endothelium and believed propagation at this site [16], the possibility that decreased PRCP may be associated with disruption of endothelial homeostasis is intriguing. The down-regulation of calgranulin C (*S100A12*) and transglutaminase 1 (*TGM1*) mRNAs also may have effects related to vascular functions. Acute infection with *M. suis* in pigs has been shown to result in disturbed endothelial homeostasis and endothelial denudation [16]. While direct effects of *M. suis* infection on the endothelial cells of pigs were not evaluated in this study, our results suggest that vascular homeostasis may be indirectly affected. It is important to note that the sources of these mRNAs in this study are nucleated cells in the peripheral blood, and its effects on endothelial cells are speculative. Nevertheless, possible changes in vascular homeostasis could have a significant effect on porcine models, such as the use of pigs to study diabetes mellitus, a metabolic disorder known to cause vascular alterations.

In summary, subclinical, possibly chronic, *M. suis* infection was capable of causing significant transcriptional alterations in host pathways commonly studied in biomedical research (e.g. innate immune response, programmed cell death, vascular homeostasis), and may severely affect scientific results using porcine models. We strongly recommend that pigs be screened for *M. suis* infection by PCR before being used in research studies.

## Additional files

**Additional file 1.** **Results of** ***Mycoplasma suis*** **microbead immunoassay (MIA).** Median Fluorescence Intensity (MFI) of *Mycoplasma* suis-infected and sham-inoculated pigs as detected in serum samples collected along the experiment. Serum antibodies were measured using recombinant GrpE (rGrpE) protein in a MIA [30]. Sham-inoculated group (control group): pig #1, pig #2 and pig #3. *M. suis*-inoculated group (infected group): pig #5, pig #6, pig #7. Day 0 corresponds to inoculation day. MFI cut-off was 74.07. It is important to note that a high cut-off may have been encountered due to the low number of animals to determine a cut-off, which increases the standard deviation. MFI values in bold correspond to positive results based on cut-off values for each group. ND: not done.

**Additional file 2.** **Results of RNA-Seq analyses.** List of differentially expressed (DE) genes identified in whole blood of *Mycoplasma suis*-infected pigs (when compared to sham-infected group) separated by all three methods of RNA-Seq analyses (DESeq2, edgeR, Cufflinks). First tab describes all DE genes (*p*-value ≤ 0.1) that were detected in all three methods, in two methods, followed by each individual method. The following tabs report the statistics of each evaluated gene for each method. Highlighted in grey are statistically significant DE genes (dark grey: *p*-value ≤ 0.05; light grey *p*-value ≤ 0.1).

**Additional file 3.** **Relative expression (RE) of differentially expressed (DE) in pigs infected with** ***Mycoplasma suis*** **validated by qRT-PCR.** Relative expression profile (*GAPDH* as reference control gene) of DE genes identified in the *M. suis*-infected pigs compared to the control group (non-infected). Validated genes are: ISG15 ubiquitin-like modifier (*ISG15*), interleukin 22 receptor, alpha 2 (*IL22RA2*), CD274 molecule (*CD274*), BCL2-like 14 (apoptosis facilitator) (*BCL2*), chemokine (C-C motif) receptor 5 (*CCR5*), CD180 molecule (*CD180*), allograft inflammatory factor 1 (*AIF1*), interleukin 15 (*IL15*), protein tyrosine phosphatase, receptor type, O (*PTPRO*), and Toll-like receptor 8 (*TLR8*). Black dots in each graphic represent the RE of a respective gene for each pig; blue vertical lines represent the standard deviation in each group. * *p*-value ≤ 0.1, and ** *p*-value ≤ 0.05.

**Additional file 4.** **Gene ontology (GO) categories of all 55 differentially expressed (DE) genes of** ***Mycoplasma suis*** **infected pigs.** First tab describes raw results of GO analysis, with all DE genes and their correspondent GO categories (and functions). Second tab shows the identified functions of the “biological process” category of all 55 DE genes and the number of reported genes for each function. Functional categories with the largest number of genes are highlighted in yellow. GO analysis was performed using Blast2Go software [39].
