# Supplementary figures and images for: RNA-Seq based transcriptome of whole blood from immunocompetent pigs (Sus scrofa) experimentally infected with Mycoplasma suis strain Illinois
Source: Vet Res. 2018 Jun 18;49:49. doi: 10.1186/s13567-018-0546-6 (PMC6006945; doi:10.1186/s13567-018-0546-6)

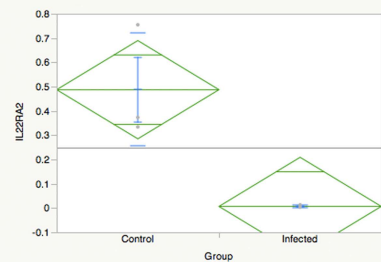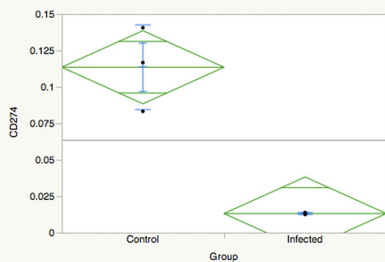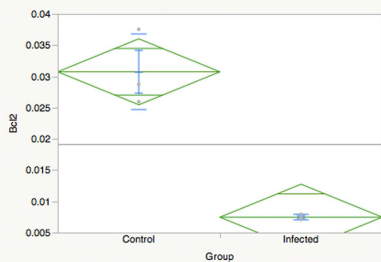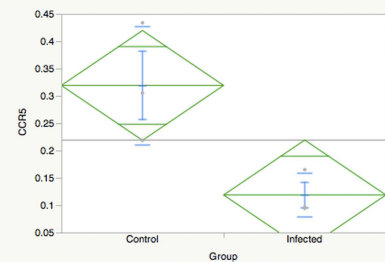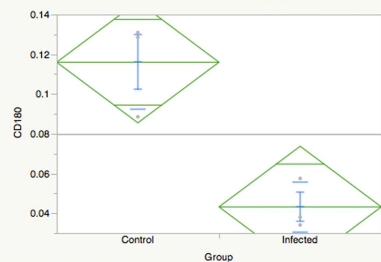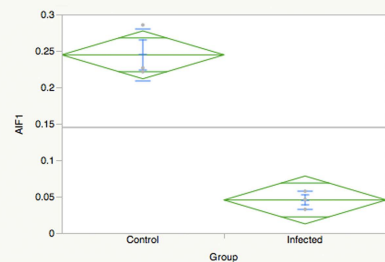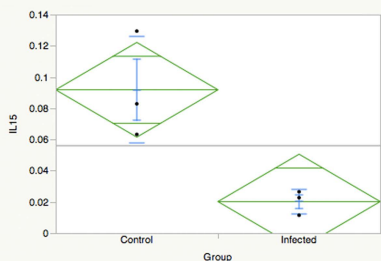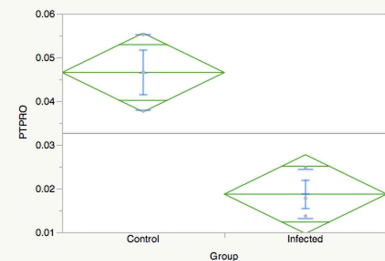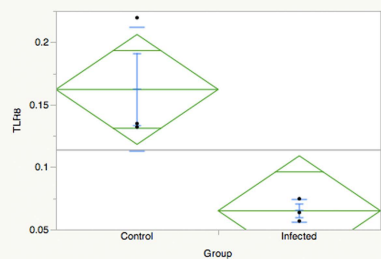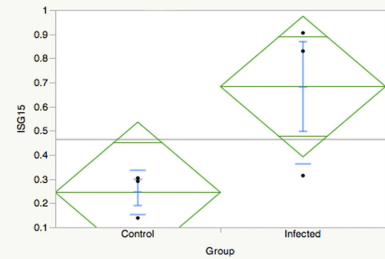

Supplement: Supplementary file 3 — Additional file 3. Relative expression (RE) of differentially expressed (DE) in pigs infected with Mycoplasma suis validated by qRT-PCR. Relative expression profile (GAPDH as reference control gene) of DE genes identified in the M. suis-infected pigs compared to the control group (non-infected). Validated genes are: ISG15 ubiquitin-like modifier (ISG15), interleukin 22 receptor, alpha 2 (IL22RA2), CD274 molecule (CD274), BCL2-like 14 (apoptosis facilitator) (BCL2), chemokine (C-C motif) receptor 5 (CCR5), CD180 molecule (CD180), allograft inflammatory factor 1 (AIF1), interleukin 15 (IL15), protein tyrosine phosphatase, receptor type, O (PTPRO), and Toll-like receptor 8 (TLR8). Black dots in each graphic represent the RE of a respective gene for each pig; blue vertical lines represent the standard deviation in each group. * p-value ≤ 0.1, and ** p-value ≤ 0.05. [file 13567_2018_546_MOESM3_ESM.pdf]
